# Dynamic Changes in Viral Loads during Co-Infection with a Recombinant Turkey Herpesvirus Vector Vaccine and Very Virulent Marek’s Disease Virus In Vivo

**DOI:** 10.3390/v16071042

**Published:** 2024-06-27

**Authors:** Tian Ding, Min Xiong, Yang Xu, Xing Pu, Qin-sen Wang, Mo-ru Xu, Hong-xia Shao, Kun Qian, Hai-bin Dang, Ai-jian Qin

**Affiliations:** 1 The Ministry of Education Key Laboratory for Avian Preventive Medicine, Yangzhou University, Yangzhou 225009, China; dt2895843407@163.com (T.D.); xiongmin669@163.com (M.X.); dz120210015@stu.yzu.edu.cn (Q.-s.W.); 008462@yzu.edu.cn (M.-r.X.); hxshao@yzu.edu.cn (H.-x.S.); qiankun@yzu.edu.cn (K.Q.); 2 Jiangsu Co-Innovation Center for Prevention and Control of Important Animal Infectious Diseases and Zoonoses, Yangzhou 225009, China; 3 College of Veterinary Medicine, Sichuan Agricultural University, Chengdu 625014, China; xuyang89xwx@sina.com; 4 Nanchang Boehringer—Ingelheim Animal Health Co., Ltd., Nanchang 330096, China; xing.pu@boehringer-ingelheim.com (X.P.); donald.dang@boehringer-ingelheim.com (H.-b.D.); 5 Joint International Research Laboratory of Agriculture and Agri-Product Safety, Ministry of Education, Yangzhou University, Yangzhou 225012, China

**Keywords:** Marek’s disease virus, Md5, rHVT-IBD vaccine, viral load

## Abstract

Marek’s disease (MD), caused by the Marek’s disease virus (MDV), is a common infectious tumor disease in chickens and was the first neoplastic disease preventable by vaccination. However, the vaccine cannot completely prevent virulent MDV infections, allowing both the vaccine and virulent MDV to coexist in the same chicken for extended periods. This study aims to investigate the changes in viral load of the very virulent strain Md5 and the rHVT-IBD vaccine in different chicken tissues using a real-time PCR assay. The results showed that the rHVT-IBD vaccine significantly reduced the viral load of MDV-Md5 in different organs, while the load of rHVT-IBD was significantly increased when co-infected with Md5. Additionally, co-infection with Md5 and rHVT-IBD in chickens not only changed the original viral load of both viruses but also affected the positive rate of Md5 at 14 days post-vaccination. The positive rate decreased from 100% to 14.29% (feather tips), 0% (skin), 33.33% (liver), 16.67% (spleen), 28.57% (thymus), 33.33% (bursa), and 66.67% (PBL), respectively. This study enhances our understanding of the interactions between HVT vector vaccines and very virulent MDV in chickens and provides valuable insights for the future development of MD vaccines.

## 1. Introduction

Marek’s disease (MD) is a contagious oncological disease that seriously affects the poultry industry worldwide. Infected chickens manifest many symptoms, including wasting, limb paralysis, mononuclear cell infiltration, and even the tumor formation of peripheral nerves, gonads, iris, and various internal organs. The infection of B/T cell lytic causes atrophy of the thymus and bursa, resulting in immunosuppression and increased susceptibility to other viruses [1]. Significantly, MD is the first oncological disease that can be prevented by vaccines. A single successful immunization of chickens with the MD vaccine provides lifelong protection [2]. The causative agent of MD is Marek’s disease virus (MDV), classified in the α Herpesviridae family, which is a cell-associated virus. The genome of MDV consists of a long unique region (UL), a short unique region (US), and also consists of long and short repeat regions [3]. MDV can be divided into three serotypes, which are MDV-1, MDV-2, and MDV-3. MDV-1 includes pathogenic MDV and attenuated MDV, such as the CVI988 and 814 strain. MDV-2 is a non-pathogenic MDV, such as SB-1, and MDV-3 is the turkey herpesvirus (HVT). According to differences in pathogenicity, MDV-1 can also be further divided into mild MDV (mMDV), virulent MDV (vMDV), very virulent MDV (vvMDV), and very virulent plus MDV (vv + MDV) [4]. The predominant mode of transmission of MDV is through the epithelial cells of the feather capsule as well as shed dander. Due to its intact viral particles, it can remain infectious at room temperature [4]. Therefore, the detection of viral load in feathers and skin is important for studying MDV shedding.

Since the first isolation of MDV in 1968 [5], the prevention and control of MD have been highly dependent on vaccination. The first commercially available vaccine, HPRS-16/Att, had limited use due to the emergence of a more effective alternative, Fc126 (HVT) [5]. With the persistent outbreaks of very virulent viruses, MD vaccines are continually updated to withstand the challenge of these virulent strains. Despite the increasing efficacy of vaccines, unfortunately, all current MD vaccines have failed to induce sterile immunity against the infection and spread of MDV. Selective pressure from MD vaccines may have caused a genetic drift in emerging strains, leading to increased virulence. [6,7,8].

The current effective MD vaccines are all live vaccines. Sylvie Remy et al. [9] demonstrated how rHVT-ND can survive in most chickens for more than 41 weeks and maintain a certain viral load. Gimeno et al. [10] showed that highly protective vaccines replicate better in chickens compared to low-protection vaccines. Consequently, monitoring vaccine load in chicken tissues, especially feather tips, has become an important method to assess the success of immunization [11,12,13,14]. It should not be overlooked that pathogenic MDV can also replicate long-term in tissues for extended periods after the infection of chickens, similar to vaccines [15]. This raises the concern that widespread vaccine usage may lead to the prolonged coexistence of vaccines and wild-type viruses in commercial chickens. The coexistence of these two viruses could potentially impact each other’s replication, which has been studied by researchers in recent years [16,17]. With the increasing popularity of “advanced vaccines”, especially HVT recombinant vaccines, the change in viral replication in chickens and their interaction with pathogenic MDV require further investigation. Our research aims to explore the dynamic viral load changes caused by the interaction between a commercial rHVT-IBD recombinant vaccine and vvMDV Md5. This study contributes to our understanding of MD vaccine development and paves the way for the future development of new MD vaccines.

## 2. Materials and Methods

### 2.1. Experimental Chickens, Vaccination, and Challenges

A total of 200 SPF chickens (white leghorn 1-day-old) were obtained from Boehringer Ingelheim (Nantong, China) and randomly divided into four groups: 35 in the control group, 55 in the vaccinated group, 55 in the challenged group, and 55 in the vaccinated/challenged group. One-day-old chickens in the vaccinated and vaccinated/challenged groups were vaccinated subcutaneously with the rHVT-IBD vaccine (VAXXITEK vaccine provided by Boehringer Ingelheim) at a dose of 5000 PFU/per chicken. At 7 days of age, chickens in the challenged and vaccinated/challenged groups were infected with vvMDV-Md5 intraperitoneally at a dose of 1000 PFU/per chicken. The virus was stored in the Key Laboratory of Avian Preventive Medicine of the Ministry of Education, Yangzhou University. The Md5-infected CEFs (in passage 40, as supported by Professor Zhizhong Cui [18]) were used for the challenge. Chickens in the control group were given a mock inoculation of the vaccine diluent (0.2 mL/bird). Tissue samples were collected on days 7, 14, 21, 28, 35, 49, and 67 post-vaccination (d.p.v.) to cover important stages of viral replication in vivo. Each sample was taken from 5 chickens in the control group and 7 chickens in the other groups (chickens in the challenge group were infected with Md5 at 7 d.p.v. and were not sampled). Feather tips, skin (from the back and legs), liver, spleen, thymus, bursa, and peripheral blood lymphocyte (PBL) were collected from chickens after euthanasia.

### 2.2. PBL Isolation and DNA Extraction

PBL was isolated from blood using the chicken PBL isolate kit (Beijing Solarbio Science & Technology Co., Ltd., Beijing, China). Fresh anticoagulated blood was taken and diluted with an equal volume of tissue diluent. An appropriate amount of diluted blood was added to the centrifuge tube, causing the diluted blood to be above the lymphocyte separation medium. After 1000× *g* of centrifugation for 30 min at room temperature, a clear stratification was formed with the uppermost layer as the diluted plasma layer, the middle layer as the transparent separating fluid layer, and the white membrane layer between the plasma and separating fluid as the lymphocyte layer. The lymphocytes were carefully aspirated into a 15 mL clean centrifuge tube with a cell washing solution at 250× *g* and were centrifuged for 10 min. Finally, the supernatant was discarded, and the cells were resuspended for DNA extraction.

DNA from tissues and PBL was extracted using the DNA/RNA Extraction Kit (Nanjing Vazyme Biotech Co., Ltd., Nanjing, China) following the manufacturer’s instructions. Briefly, A portion of the tissue was taken and added to PBS for grinding. In total, 200 μL of tissue homogenate/PBL and 20 μL of Proteinase K were added to the kit. We placed the kit into the Vazyme VNP-32P nucleic acid extractor for automated extraction. The obtained DNA solution was stored at −20 °C and used for real-time PCR.

### 2.3. Primers and Real-Time PCR

The primers and probes were designed according to the previous report [19]. Briefly, the qPCR method distinguished between the two viruses in tissues by detecting the MDV type 1 pathogenic strain-specific gene *pp*38 and the HVT-specific gene *SORF*1. The sequences of the primers and probes were similar to the reference [19]. A real-time PCR was performed using Roche LightCycler 96 following the reaction system, with a total mixture of 20 μL as follows: Premix Ex Taq™ (Probe qPCR) 10 μL, 0.08 μL of each primer (100 μM), 0.04 μL of each probe (100 μM), 2 μL of viral DNA and 7.4 μL of ddH_2_O was added to make up the reaction system. The amplification conditions were set in pre-incubation at 95 °C for 30 s, with 2-step amplification at 95 °C for 5 s and 60 °C for 30 s (40 cycles), and cooling at 37 °C for 30 s. The fluorescence signal was determined at the end of each cycle of the 60 °C extension step. According to the chicken internal reference gene *Ovo*-designed primers and probes, the number of cells was quantified by the copy number (double copy gene) of the internal reference gene *Ovo* according to the research of Rémy [20], and the copy number of the virus was quantified per 10^6^ cells in each sample.

### 2.4. Data Analyses

GraphPad Prism v9.0 was used to plot standard graphs, including line charts and scatter plots. An unpaired *t*-test was applied for statistical analysis. All data were reported as mean ± S.E.M, with a *p*-value < 0.05 considered significant.

## 3. Result

### 3.1. Mortality and Morbidity of Chickens

**Figure 1:**
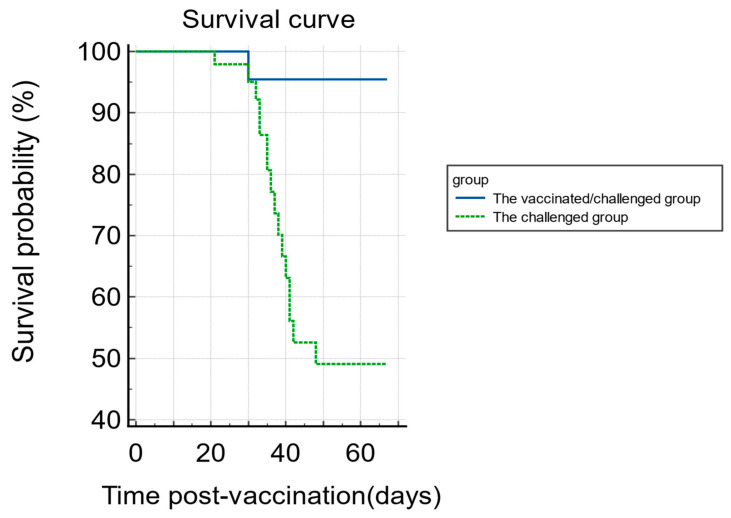
Survival probabilities were counted based on the number of deaths per day after rHVT-IBD vaccination in the challenged group and the vaccinated/challenged groups. Due to the need to collect tissue samples at six or seven time points, euthanized chickens were not included in the statistics to facilitate the presentation of deaths in both groups.

The mortality and morbidity of chickens were monitored to examine the success of the Md5 challenge and rHVT-IBD immunization. In the challenged group (Table 1), 16 chickens died and had tumor lesions. Chickens in the challenged group that died at 21 d.p.v. (1 chicken) and at 35 d.p.v. (2 chickens) were included in the tissue sampling since they died on the sampling timepoints. The other 13 chickens that died between 30 and 34 d.p.v. and 36 and 48 d.p.v. were not included in the sampling. Five additional chickens in the challenged group were found to have tumor lesions upon euthanasia at sampling timepoints (one at 49 d.p.v. and four at 67 d.p.v.). In the vaccinated/challenged group (Table 2), only 1 chicken died at 30 d.p.v. and it had tumor lesions. One chicken at 67 d.p.v. in the vaccinated/challenged group had tumor lesions upon sampling. Chickens randomly euthanized for sampling were considered censored animals and not included in the mortality statistics. The survival probability of chickens in the challenged and the vaccinated/challenged groups is shown by the Kaplan–Meier survival curve (Figure 1). There were no deaths or typical lesions of Marek’s disease in the vaccinated group and the control group.

### 3.2. The Load Patterns of rHVT-IBD and Md5 in Different Organs

**Figure 2:**
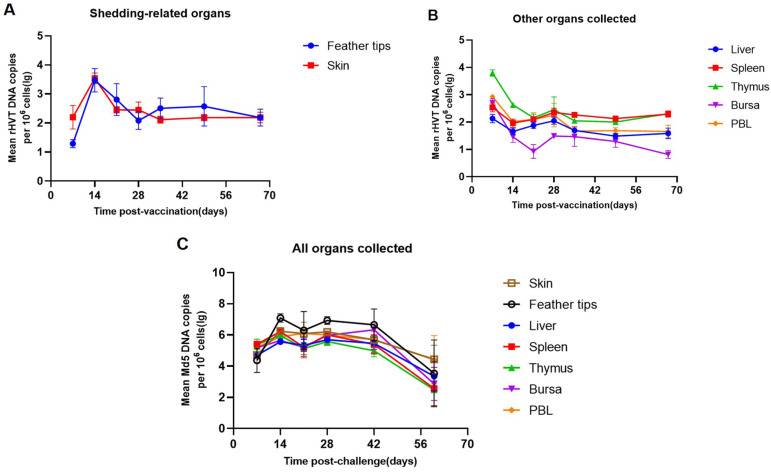
Viral loads of the rHVT-IBD vaccine were detected in chicken organs of the vaccinated groups collected at seven time points (7, 14, 21, 28, 35, 49, and 67 d.p.v.). The Md5 viral load in chicken organs was detected at six time points in the challenged group (7, 14, 21, 28, 42, 60 d.p.v.). DNA was extracted from each organ, and real-time PCR was performed to detect the vaccine load. The following was ascertained: (**A**), the viral load change in the rHVT-IBD vaccine in feather tips and skin; (**B**), the viral load change in the rHVT-IBD vaccine in liver, spleen, thymus, bursa, PBL; and (**C**), the viral load change in Md5 in all organs collected. Each point corresponds to a sampling time point, *n* = 7, and the viral load of rHVT-IBD was normalized per million cells. Error bars indicate S.E.M.

To analyze the dynamic load changes in the rHVT-IBD and Md5 in different tissues, the viral loads of rHVT-IBD in feather tips, skin, liver, spleen, thymus, bursa, and PBL at 7–67 days post-vaccination (d.p.v.) were examined by a real-time PCR. The loading changes in the rHVT-IBD vaccine in feather tips and skin (7–28 d.p.v.) showed similar patterns of vaccine loading (Figure 2A). Conversely, the patterns of rHVT-IBD loading in organs, including the liver, spleen, thymus, bursa, and peripheral blood lymphocytes, were similar to one another but differed from those in feather tips and skin (Figure 2B). Specifically, the rHVT-IBD genome in feather tips and skin was quantifiable at 7 d.p.v. and reached a peak level at 14 d.p.v., followed by a decrease to 28 d.p.v. afterward; the viral load of rHVT-IBD remained at the same level at 28–67 d.p.v. Interestingly, the load of rHVT-IBD demonstrated a “W” pattern in other organs, where the viral load of the vaccine was already at a high level at 7 d.p.v., then significantly decreased at 14 d.p.v., followed by an increase at 28 d.p.v., and decline at 35 d.p.v., before finally increasing slightly at 67 d.p.v. Md5 showed similar changes in all organs collected (Figure 2C). At each sampling time point, the viral load of Md5 was higher compared to rHVT. The changes in Md5 viral load in all tissues followed an almost identical pattern, with an increase in viral load from 7–14 days post-challenge, a slight decrease from 14–21 days post-challenge, and a rebound at 28 days post-challenge. After 42 days post-challenge, the viral load appeared to decline, but the Md5 load was still detectable until the last sampling.

### 3.3. rHVT-IBD Can Reduce Md5 Load In Vivo

**Figure 3:**
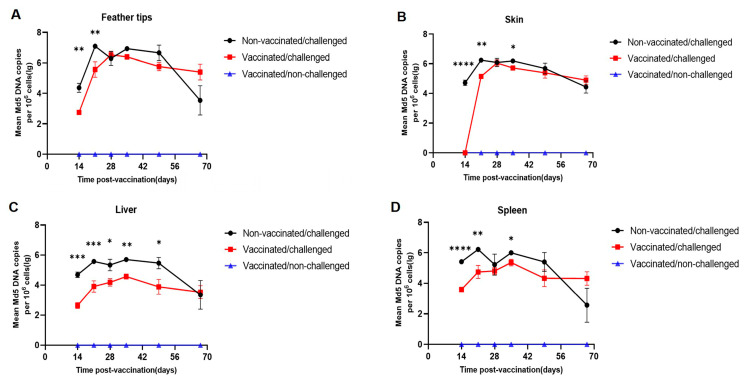

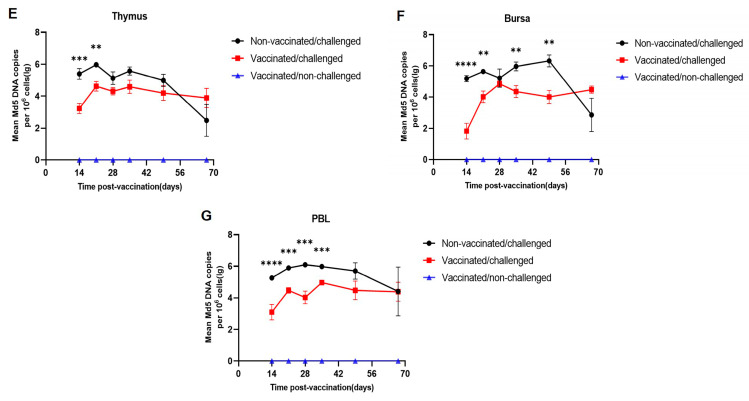
Viral loads of the rHVT-IBD vaccine were detected in chicken organs of the challenged and vaccinated/challenged groups collected at six time points (14, 21, 28, 35, 49, and 67 d.p.v.). DNA was extracted, and a real-time PCR was performed to detect the viral load of Md5. The following was ascertained: (**A**), load changes in Md5 in feather tips; (**B**), load changes in Md5 in the skin; (**C**), load changes in Md5 in the liver; (**D**), load changes in Md5 in the spleen; (**E**), load changes in Md5 in the thymus; (**F**), load changes in Md5 in bursa; and (**G**), load changes in Md5 in PBL. The black line represents the Md5 load changes in the challenged group, and the red line represents the load changes in the vaccinated/challenged group. Each dot corresponds to a sampling time point, *n* = 7, and the viral load of Md5 was normalized per million cells. The results of the Md5 viral load between the challenged and the vaccinated/challenged groups were statistically analyzed using an unpaired *t*-test, and all data were reported as the mean ± S.E.M when a *p*-value < 0.05 was considered significant. * *p* < 0.05, ** *p* < 0.01, *** *p* < 0.001, **** *p* < 0.0001.

To investigate the effect of the rHVT-IBD vaccine on Md5 loading, the dynamic load changes in Md5 in the organs of chickens in the challenged and vaccinated/challenged groups were examined by real-time PCR. The results showed that rHVT-IBD could reduce the load of pathogenic MDV by approximately 100-fold in chickens at 14 d.p.v. (Figure 3A–G). It was interesting that the most significant suppression during this early stage of immunity was in the skin, which is an organ for virus shedding. None of the seven skin samples collected from the vaccinated/challenged group showed any detectable Md5 load at 14 d.p.v., indicating that the rHVT-IBD vaccine could inhibit the Md5 load by nearly 10,000-fold at that time point. However, this inhibitory effect of HVT decreased significantly over time, while no significant difference was found in the Md5 load in all organs of chickens from the challenged and vaccinated/challenged groups at 67 days post-vaccination.

### 3.4. Md5 Increases rHVT-IBD Load In Vivo

**Figure 4:**
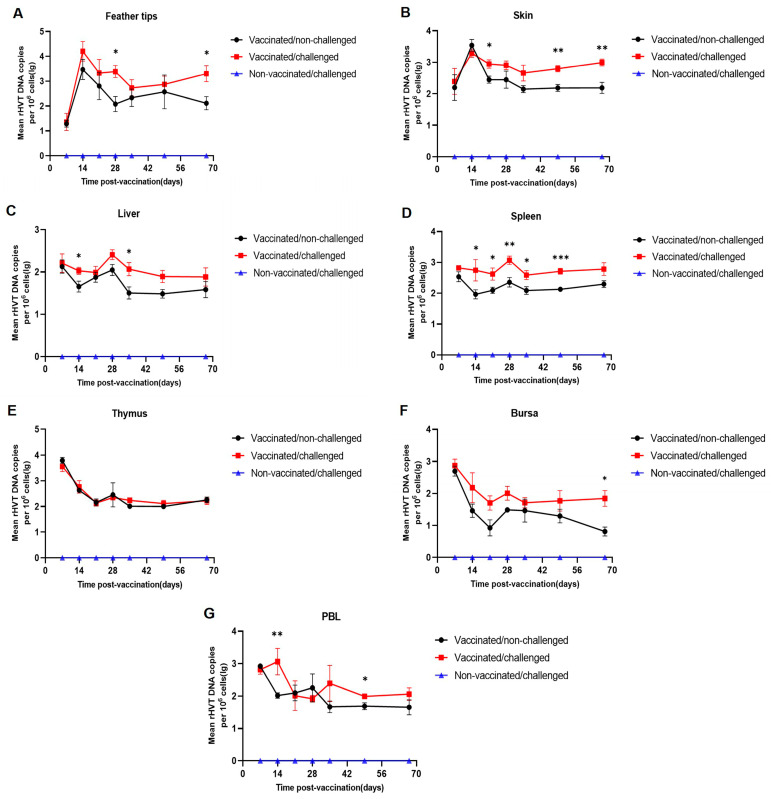
Viral loads of the rHVT-IBD vaccine were detected in chicken organs of the vaccinated and vaccinated/challenged groups collected at seven time points (7, 14, 21, 28, 35, 49, and 67 d.p.v.). DNA was extracted, and a real-time PCR was performed to detect the viral load of the vaccine. The following was observed: (**A**), load in of rHVT-IBD in feather tips; (**B**), load changes in rHVT-IBD in skin; (**C**), load changes in rHVT-IBD in the liver; (**D**), load changes in rHVT-IBD in the spleen; (**E**), load changes in rHVT-IBD in the thymus; (**F**), load changes in rHVT-IBD in bursa; and (**G**), load changes in rHVT-IBD in PBL. The black line represents the load changes in rHVT-IBD in the vaccinated group, and the red line represents the load changes in rHVT-IBD in the vaccinated/challenged group. Each point corresponds to a sampling time point, *n* = 7, and the viral load of rHVT-IBD was normalized per million cells. The results of the rHVT-IBD viral load between the vaccinated and the vaccinated/challenged groups were statistically analyzed using an unpaired *t*-test, and all data were reported as the mean ± S.E.M when a *p*-value <0.05 was considered significant. * *p* < 0.05, ** *p* < 0.01, *** *p* < 0.001.

To investigate the effect of Md5 on the load of the rHVT-IBD vaccine, we compared the dynamic load changes in the rHVT-IBD vaccine between the vaccinated and the vaccinated/challenged groups (Figure 4A–G). There was no significant difference in vaccine loads across all organs of chickens between the vaccinated and vaccinated/challenged groups at 7 d.p.v. without challenge. The rHVT-IBD vaccine showed some increase in load levels in response to Md5, with feather tips, skin, liver, spleen, bursa, and PBL showing significant increases at several sampling time points. The most significant increase occurred in the spleen at 14–49 d.p.v. This differed from the change in Md5 loading under the influence of rHVT-IBD. Compared to vaccine loads in other organs, vaccine loads in the thymus of chickens in the vaccinated group did not differ from those in the vaccinated/challenged group at all sampling time points (7–67 d.p.v.). (Figure 3E). Furthermore, we observed that this increase in rHVT-IBD loading did not disappear over time. At 67 d.p.v., the vaccine load of feather tips, skin, and bursa was still significantly higher in the vaccinated/challenged group than in the vaccinated group. This was distinct from the phenomenon of reduced Md5 loads.

### 3.5. Coinfection of Md5 and rHVT-IBD Reduces the Positive Rate of Md5

**Figure 5:**
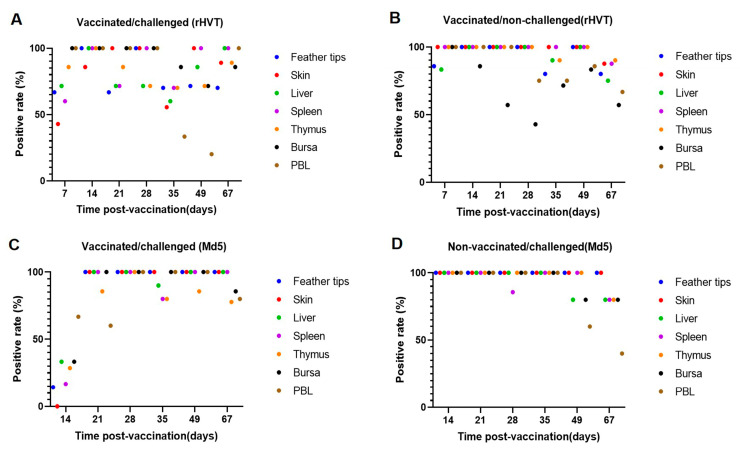
The organs of chickens from the vaccinated/challenged group and vaccinated group were collected at seven time points (7, 14, 21, 28, 35, 49, 67 d.p.v.). The challenged group’s organs were collected at six time points (14, 21, 28, 35, 49, 67 d.p.v.). DNA was extracted, and a real-time PCR was performed to detect Md5 and rHVT-IBD replication. According to the total number of samples per group at each time point (*n* = 7), the positive rate of Md5 and rHVT-IBD in each organ at each time point was calculated, and the dots of different colors corresponded to different organs. The results showed (**A**), the positive rate of rHVT-IBD in the vaccinated/challenged group; (**B**), the positive rate of rHVT-IBD in the vaccinated group; (**C**), the positive rate of Md5 in the vaccinated/challenged group; and (**D**), the positive rate of Md5 in the challenged group.

Analyzing the viral load of each organ, we found that viral load was undetectable in some samples, consistent with the findings of Gimeno et al. [21]. To further investigate the effect of the interaction between Md5 and rHVT-IBD on viral load, we repeated the testing of these samples to rule out the possibility of errors in DNA extraction and other procedures. It was found that the co-infection of Md5 and rHVT-IBD in chickens not only changed the original viral load of Md5 but also affected the positive rate, particularly at 14 d.p.v. (Figure 5C). The virus-positive rate in the organs of chickens in the vaccinated and challenged groups did not reach 100% (Figure 5B,D). However, in the vaccinated/challenged group, the positive rate of Md5 was further reduced due to the influence of another virus (Figure 5C). This reduction in viral positive rate was not confined to a single organ but was observed across all organs. Moreover, the most significant reduction in the positive rate occurred at the onset of the challenge (14 d.p.v.), when the positive rate of Md5 was severely impacted in all seven types of organs collected. The positive rate reduced from 100% to 14.29% (feather tips), 0% (skin), 33.33% (liver), 16.67% (spleen), 28.57% (thymus), 33.33% (bursa), and 66.67% (PBL), respectively. rHVT-IBD vaccination significantly reduced its viral load in organs during the pre-infection period of Md5 (14–21 d.p.v.). It also markedly diminished the Md5 positive rate, leading to an undetectable viral load.

## 4. Discussion

MD vaccines have played an important role in protecting chickens against tumor lesions and immunosuppression induced by pathogenic MDV. While Lee et al. [22] concluded that the Meq gene deletion vaccine provided good protection, most people still believe that the most effective MD vaccine (immunization alone) is CVI988/Rispens [23,24]. With the successful development of recombinant HVT vector vaccines, HVTs have been used worldwide. Broiler chickens are usually vaccinated with rHVT alone, while rHVT + CVI988 [25] is often used with others. The immune efficacy of the MD vaccine is affected by the age of chickens and vaccine dose [26,27], while also related to the replication of the vaccine in vivo. Currently, there have been some studies on the replication of MD vaccines in vivo, but few works of research exist on rHVT vaccines. Gimeno et al. [21] found that rHVT-LT replicated more slowly than HVT in vivo.

In this study, we detected dynamic viral load changes in rHVT-IBD and Md5 in different organs of chickens by a real-time PCR. Changes in loadings of the rHVT-IBD vaccine in shedding-related organs (feather tips and skin) differed from those in other organs. However, this difference did not appear in the Md5-challenged group. Chickens immunized with/without the rHVT-IBD vaccine were challenged with vvMDV-Md5 (1000 PFU) at 7 days of age. It was found that the vaccination of rHVT-IBD reduced the Md5 load in vivo, which was similar to the inhibitory effect of CVI988/Rispens [28,29], SB-1 [16], and HVT on RB1B [30]. It will be very interesting to follow up on whether the transformed T cells are associated with the Md5 load. In addition, a very low viral load of Md5 was detected in the skin of chickens from the vaccinated/challenged group compared to the challenged group at 14 d.p.v., indicating that vaccine rHVT-IBD significantly inhibited Md5 load. Similarly, the viral load of Md5 in other organs of chickens in the vaccinated/challenged group tended to be lower compared to those in the challenged group due to the effect of rHVT-IBD at 14 d.p.v. We examined the effect of Md5 on the load of rHVT-IBD. The results showed that the load of rHVT-IBD was significantly higher in chickens from the vaccinated/challenged group than in the challenged group at some time points, which was consistent with previous studies on the SB-1, BAC SB-1 [16], and HVT [17,30] vaccines. However, we did not find any increase in the vaccine load in the thymus at any time point. Notably, the infection of virulent MDV had no significant effect on the viral load of CVI988/Rispens in the organs of CVI988-vaccinated chickens [28]. Haq et al. [31] found that the interaction of two kinds of MDV in vivo promoted the replication of one or both. This phenomenon occurred not only in the co-infection of two MDV strains but also in co-infection with other viruses, such as the co-infection of MDV and ALV, leading to an increase in ALV replication [32], and co-infection of Porcine circovirus type 2(PCV-2) with Porcine Reproductive and Respiratory Syndrome Virus(PRRSV) promoting PCV-2 replication [33]. The co-infection of two viruses in vivo may be a symbiotic or competitive relationship. Dunn et al. [9] believed that the changes in this replication may be partly determined by MDV virulence after studying the competition of two virulent MDVs in vivo. Du et al. [34,35] demonstrated that the co-infection of MDV and REV via RIOK3-mediated Akt phosphorylation can promote the replication of MDV and REV. However, due to the gradual reduction in the number of chickens in each group as a result of euthanasia during sampling, we only calculated the disease incidence rate among those who survived until 67 days. The insufficient sample size represents a weakness in this experiment, which we will address in the future.

By comparing the change in viral positivity of the two viruses in organs, it was found that the co-infection with rHVT-IBD and Md5 not only affected the viral load but also reduced the positive rate of Md5. However, there are numerous factors that can influence the rate of vaccine positivity, which are related to the vaccination method. Gimeno et al. [21,23] concluded that the ovo vaccination of chicken embryos during ED17-ED19 can increase the positive rate of vaccination. This could explain why the vaccine-positive rate was not 100% for chickens in the vaccinated and vaccinated/challenged groups.

Current MD vaccines cannot prevent infection by wild-type MDV, and the co-replication of the vaccine and wild-type MDV in chickens remains a challenging issue to address. Xu et al. [36] expressed concerns regarding the genetic recombination of the two viruses to produce a new strain. Regrettably, these concerns have now materialized. Zhang et al. [37] isolated six strains of wild MDV from commercial flocks that were recombined from the CVI988 skeleton with the virulence strain’s partial unique short region. The recombinant strains were weakly virulent but showed temporal damage to immune organs. This suggests that the recombinant mutation of MDV vaccine strains with virulent strains could be a significant risk that cannot be ignored. Lantier et al. [38] demonstrated by in vivo bioluminescence imaging that the rostrum and foot skin of chickens are also sites of MDV replication and shedding. Much of the load changes in MDV in vivo after infection require further investigation, and we believe that a comprehensive understanding of vaccine and pathogenic MDV load changes in chickens will be useful for the further development of MD vaccines.

## Figures and Tables

**Table 1:** Survival probability of chickens in the challenged group.

Survival Time (t)	Number of Individuals with Events (m)	Censored (q)	Number of Survivors (*n*)	(*n*-m)/*n*	Survival Probability S(t)
7 d.p.v.	0	0	55	1	100%
14 d.p.v.	0	7	55	1	100%
21 d.p.v.	1	6	48	0.9792	97.92%
28 d.p.v.	0	7	41	1	97.92%
30 d.p.v.	1	0	34	0.9706	95.04%
32 d.p.v.	1	0	33	0.9697	92.16%
33 d.p.v.	2	0	32	0.9375	86.40%
35 d.p.v.	2	5	30	0.9333	80.64%
36 d.p.v.	1	0	23	0.9565	77.13%
37 d.p.v.	1	0	22	0.9545	73.62%
38 d.p.v.	1	0	21	0.9524	70.12%
39 d.p.v.	1	0	20	0.95	66.61%
40 d.p.v.	1	0	19	0.9474	63.11%
41 d.p.v.	2	0	18	0.8889	56.10%
42 d.p.v.	1	0	16	0.9375	52.59%
48 d.p.v.	1	0	15	0.9333	49.08%
49 d.p.v.	0	7	14	1	49.08%
67 d.p.v.	0	7	7	1	49.08%

**Table 2:** Survival probability of chickens in the vaccinated/challenged group.

Survival Time (t)	Number of Individuals with Events (m)	Censored (q)	Number of Survivors (*n*)	(*n*-m)/*n*	Survival Probability S(t)
7 d.p.v.	0	7	55	1	100%
14 d.p.v.	0	7	48	1	100%
21 d.p.v.	0	7	41	1	100%
28 d.p.v.	0	7	34	1	100%
30 d.p.v.	1	0	27	0.9629	96.29%
35 d.p.v.	0	7	26	1	96.29%
49 d.p.v.	0	7	19	1	96.29%
67 d.p.v.	0	7	12	1	96.29%

## Data Availability

All data are included in the manuscript.
